# A Pilot Randomized Trial of Oral Magnesium Supplementation on Supraventricular Arrhythmias

**DOI:** 10.3390/nu10070884

**Published:** 2018-07-10

**Authors:** Pamela L. Lutsey, Lin Y. Chen, Anne Eaton, Melanie Jaeb, Kyle D. Rudser, James D. Neaton, Alvaro Alonso

**Affiliations:** 1 Division of Epidemiology & Community Health, School of Public Health, University of Minnesota, 1300 South 2nd Street, Suite 300, Minneapolis, MN 55454, USA; jaebx008@umn.edu; 2 Cardiovascular Division, Department of Medicine, University of Minnesota, Minneapolis, MN 55455, USA; chenx484@umn.edu; 3 Division of Biostatistics, School of Public Health, University of Minnesota, Minneapolis, MN 55455, USA; eato0055@umn.edu (A.E.); rudser@umn.edu (K.D.R.); jim@ccbr.umn.edu (J.D.N.); 4 Department of Epidemiology, Rollins School of Public Health, Emory University, Atlanta, GA 30322, USA; alvaro.alonso@emory.edu

**Keywords:** magnesium, atrial fibrillation, glucose, randomized controlled trial

## Abstract

Low magnesium may increase the risk of atrial fibrillation. We conducted a double-blind pilot randomized trial to assess adherence to oral magnesium supplementation (400 mg of magnesium oxide daily) and a matching placebo, estimate the effect on circulating magnesium concentrations, and evaluate the feasibility of using an ambulatory heart rhythm monitoring device (ZioPatch) for assessing premature atrial contractions. A total of 59 participants were randomized; 73% were women, and the mean age was 62 years. A total of 98% of the participants completed the follow-up. In the magnesium supplement group, 75% of pills were taken, and in the placebo group, 83% were taken. The change in magnesium concentrations was significantly greater for those given the magnesium supplements than for those given the placebo (0.07; 95% confidence interval: 0.03, 0.12 mEq/L; *p* = 0.002). The ZioPatch wear time was approximately 13 of the requested 14 days at baseline and follow-up. There was no difference by intervention assignment in the change in log premature atrial contractions burden, glucose, or blood pressure. Gastrointestinal changes were more common among the participants assigned magnesium (50%) than among those assigned the placebo (7%), but only one person discontinued participation. In sum, compliance with the oral magnesium supplementation was very good, and acceptance of the ZioPatch monitoring was excellent. These findings support the feasibility of a larger trial for atrial fibrillation (AF) prevention with oral magnesium supplementation.

## 1. Introduction

Atrial fibrillation (AF) is a common cardiac arrhythmia characterized by irregular atrial electrical activity. In the United States (US), more than 3 million individuals had AF in 2010, and this figure is expected to more than double by 2050 [1,2,3]. Current AF treatments, including antiarrhythmic drugs and catheter ablation for rhythm restoration and oral anticoagulation for the prevention of thromboembolism, have suboptimal efficacy and carry significant risks [4]. The limitations of the available therapeutic approaches highlight the need for primary prevention interventions [5,6]. As highlighted in a 2009 National Heart, Lung, and Blood Institute (NHLBI) report [5] and stressed in a more recent Heart Rhythm Society-sponsored whitepaper [6], there is an urgent need to identify new and effective strategies for the primary prevention of AF.

Compelling evidence from numerous lines of inquiry suggests that low concentrations of serum magnesium may be causally associated with AF risk. First, magnesium supplementation is recommended as prophylaxis for the prevention of AF in patients undergoing cardiac surgery. A recent Cochrane systematic review and meta-analysis of randomized trials assessing the efficacy of magnesium supplementation for AF prevention in heart surgery reported an odds ratio of 0.55 (95% CI: 0.41, 0.73) for AF or supraventricular arrhythmia, comparing the magnesium intervention to the control [7]. Second, indirect evidence from three prospective epidemiologic studies provides some support for such intervention; each reported that individuals in the lowest versus the highest quantile of serum magnesium were 35–50% more likely to develop incident AF, after multivariable adjustment [8,9,10]. Finally, additional evidence for the effect of magnesium on the risk of arrhythmias is provided by a study of dietary magnesium restriction, in which 3 out of 14 women fed a low-magnesium diet developed AF, which resolved quickly after magnesium repletion [11].

Whether magnesium supplementation could have a role in the prevention of AF in the community has not been tested. Were magnesium supplementation shown to prevent AF and be safe over the long-term, it would be an ideal intervention for primary prevention, as it is easy to implement, inexpensive, and low concentrations are common. The population prevalence of low magnesium is not known but is believed to be high. Individuals at particularly high risk of hypomagnesemia are alcoholics, those who take certain diuretics, those with poorly controlled diabetes [12], and the elderly. In a study of nursing home residents, 33% were clinically deficient [13]. Intake of magnesium in the US population is also low, to the extent that the 2015 Dietary Guidelines Advisory Committee classified magnesium as a “shortfall nutrient”, based on the finding that approximately 50% of Americans consume less than the estimated average requirement [14]. However, dietary magnesium intake and serum magnesium are poorly correlated; in the community-based Atherosclerosis Risk in Communities (ARIC) study, the Pearson’s correlation coefficient was only 0.04 [9].

As part of the planning effort for a large randomized trial to prevent AF with magnesium supplementation, we conducted a double-blind, placebo-controlled randomized clinical trial of oral magnesium supplementation to assess supplement adherence, the side effects, the effect on serum magnesium concentration, and the feasibility of using an ambulatory monitoring device for the identification of arrhythmias.

## 2. Materials and Methods

The study was registered at Clinicaltrials.gov (# NCT02837328). The study protocol was approved by the University of Minnesota Institutional Review Board, and all participants provided written informed consent.

### 2.1. Study Participants

Participants of 55 years of age or older were recruited using fliers, the University of Minnesota StudyFinder website, invitations to individuals enrolled in the ResearchMatch research volunteer database, and invitations to University of Minnesota School of Public Health employees. The exclusion criteria included a prior history of heart disease (coronary heart disease, heart failure, AF), stroke, or known kidney disease; the use of type I or III antiarrhythmic drugs or digoxin; the current use of magnesium supplements; any prior history of allergy or intolerance to magnesium; lactose intolerance; and a prior history of inflammatory bowel disease or any severe gastrointestinal disorder. The use of multivitamins was allowable, because these typically contain relatively low dosages of Magnesium (e.g., 50 mg).

The eligible participants attended a baseline visit where measurements were conducted and a Zio^®^ XT Patch (ZioPatch; iRhythm Technologies, Inc., San Francisco, CA, USA) heart rhythm monitor was applied by trained staff. After wearing the ZioPatch for 2 weeks, the participants were randomized 1:1 to either 400 mg of magnesium oxide or a placebo using block randomization within two strata of age (younger than 65 and 65 and older). The randomization was carried out separately for the two randomization strata. In each group, a randomization schedule was generated using randomly permuted blocks of random sizes. Block sizes of 2, 4, or 6 were permitted. The randomization was implemented using the blockrand package in R.

**Figure 1:**
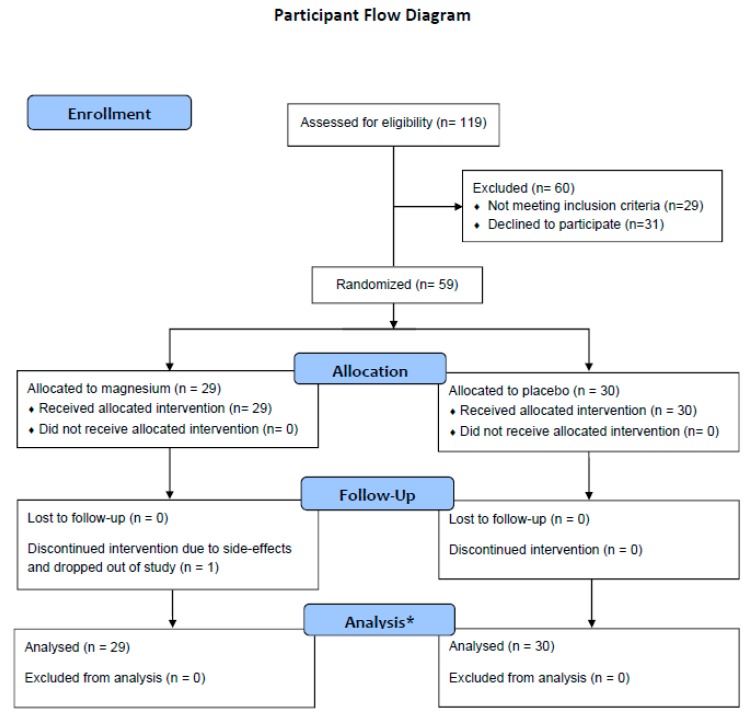
* Due to missing data on individual items, the total numbers of observations included in the linear models are 57, 57, 54, 54, 58, and 58 for the outcomes log premature atrial contraction (PAC) burden, PAC burden, serum magnesium, serum glucose, systolic blood pressure, and diastolic blood pressure, respectively.

Following randomization, the participants were mailed the study intervention, which they took for a total of 12 weeks. Then, 10 weeks after beginning the study intervention, the participants took part in a follow-up clinic visit, and a second ZioPatch was applied. The participants continued the study intervention until the second ZioPatch was removed (2 weeks after the follow-up clinic visit). A participant flow diagram is provided in Figure 1.

### 2.2. Study Intervention and Blinding

The University of Minnesota Institute for Therapeutics Discovery and Drug Development produced the active study intervention (400 mg of magnesium oxide) and the matched placebo (lactose) according to Good Manufacturing Practices. The University of Minnesota Investigational Drug Service managed the bottling per the randomization scheme. The study participants and all the study staff were blinded to the treatment given.

### 2.3. Measurements

At the baseline and follow-up clinic visits, the participants completed questionnaires, and trained study staff conducted physiological measurements (i.e., anthropometry, blood pressure), phlebotomy, and applied the ZioPatch device. Treatment compliance was assessed by a pill count at the follow-up visit. At intervention days 21, 42, and 80, the participants were also emailed unique links to online questionnaires, administered via REDCap [15], which queried compliance and asked the following open-ended question about adverse effects: “Since starting the study, have you experienced anything out of the ordinary?” Participant blinding was also assessed on intervention day 80, the last day of the study.

The ZioPatch was used to identify premature atrial contractions (PACs). PACs are supraventricular arrhythmias associated with the future risk of AF [6,16,17] and are considered an intermediate phenotype of the arrhythmia, reflecting the underlying cardiac substrate that facilitates the development of AF [18]. The participants were asked to wear the ZioPatches for 2 weeks after each clinic visit. The information obtained from the ZioPatch devices was processed by the ZEUS algorithm, a comprehensive system that analyzes electrocardiographic data received from the device [19]. We counted as PACs isolated supraventricular ectopic beats, supraventricular ectopic couplet total count, and supraventricular ectopic triplet total count. The total PACs were then divided by the number of hours the ZioPatch recorded analyzable data, which yielded PACs per hour.

The participants were asked to fast for 8 h prior to blood draws. Serum magnesium and glucose were measured using the Roche Cobas 6000 at the University of Minnesota Advanced Research and Diagnostic Laboratory. Blood pressure was measured with the participant sitting, after a 5 min rest, with a random zero sphygmomanometer (Omron Digital Blood Pressure Monitor HEM-907XL; Omron Healthcare Inc., Kyoto, Japan). Three measurements were taken; all three measurements were averaged for use in analyses. Height and weight were measured with the participants in light clothing and shoes removed. Height was measured with a research stadiometer and weight with a scale.

### 2.4. Statistical Analysis

The goal of the pilot study was to assess adherence to the magnesium supplement and the feasibility of using the ZioPatch and to collect preliminary data on PACs, a predictor of AF. The targeted sample size of 60 was determined to detect a difference in the change in log PACs (follow-up minus baseline) between treatment groups of 0.79 standard deviation units with 80% power and 5% type I error (2-sided), assuming five participants would not complete the follow-up.

All analyses were intent-to-treat. Descriptive statistics are provided according to treatment assignment for baseline characteristics, adherence, magnesium concentrations, and other outcomes. The differences in baseline characteristics between groups were assessed using *t*-tests for continuous variables and Fisher’s exact tests for categorical variables. Linear regression was used to evaluate whether change in outcomes differed according to treatment assignment, adjusting for the randomization stratification factor (age ≥65 vs. <65) and the baseline value of the outcome with robust variance estimates for confidence intervals and *p*-values. Post-hoc sensitivity analyses further adjusted for sex. As PAC burden is highly skewed, we pre-specified using log PAC burden for analysis and reported the ratio of geometric means. Pre-specified subgroup analyses were also performed, stratified by baseline magnesium concentration (< vs. ≥median). A two-sided *p*-value of <0.05 was used to indicate statistical significance. The analyses were conducted using R [20] version 3.4.0 (R Foundation, Vienna, Austria).

## 3. Results

### 3.1. Study Participants

Between March and June 2017, 59 participants were randomized: 29 were assigned to the magnesium supplement and 30 to the matching placebo. The participant characteristics were generally similar by treatment group, with the notable exception of sex; 86.2% of the participants in the treatment group were women, while in the placebo group, 60.0% were female (Table 1). The mean age of the participants was 61.5 ± 5.2 years. The baseline serum magnesium concentration was 1.74 ± 0.11 mEq/L in the participants assigned the magnesium supplements and 1.71 ± 0.10 in those assigned the placebo; 6.9% had magnesium concentrations below the threshold for clinical deficiency (<1.5 mEq/L), while 37.9% had concentrations below the threshold for subclinical deficiency (<1.7 mEq/L).

Log PAC burden (episodes per hour) at baseline was 1.26 ± 1.35 in the treatment group and 1.15 ± 1.42 in the placebo group. At baseline, the average ZioPatch analyzable time in the intervention and placebo groups were 13.1 ± 1.7 and 12.9 ± 2.6 days, respectively, with 93.1% assigned to magnesium and 90.0% assigned to placebo wearing ≥12 days.

### 3.2. Follow-Up

A total of two participants, both in the intervention group, were missing ZioPatch information at follow-up; one participant dropped out of the study, and one was missing information due to a device malfunction.

### 3.3. Adherence and Magnesium Concentrations

Based on pill count, the participants in the magnesium group took 75.1% ± 17.8% of tablets, whereas those in the placebo group took 83.4% ± 5.9%. Self-reported information about the percent of missing pills and the reasons for missing pills is provided in Table 2. Of the participants, 60% in the Magnesium group reported missing at least 1 pill, as did 52% in the placebo group. The most common reason for missing pills was forgetting. However, five individuals in the Magnesium group marked the response “makes me sick” as the reason for not taking a pill, whereas no individuals in the placebo group reported missing pills for that reason.

Over the 12-week follow-up period, those assigned magnesium supplementation had a significant increase in serum magnesium concentration as compared with those assigned the placebo (0.07 mEq/L; 95% CI: 0.03, 0.12; *p* = 0.002) (Table 3). In subgroup analyses, the change in magnesium concentration did not vary by baseline magnesium concentration (Table 4; *p*-interaction 0.24). Specifically, among the participants who at baseline were below the median serum magnesium concentration (i.e., 1.74 mEq/L), the effect of the magnesium versus the placebo on the change in the serum magnesium concentration was 0.05 (95% CI: 0.00, 0.10), whereas among those at or above the median at baseline, the effect was 0.12 (95% CI: 0.04, 0.20).

### 3.4. Effect of Magnesium Supplementation on Trial Outcomes

**Figure 2:**
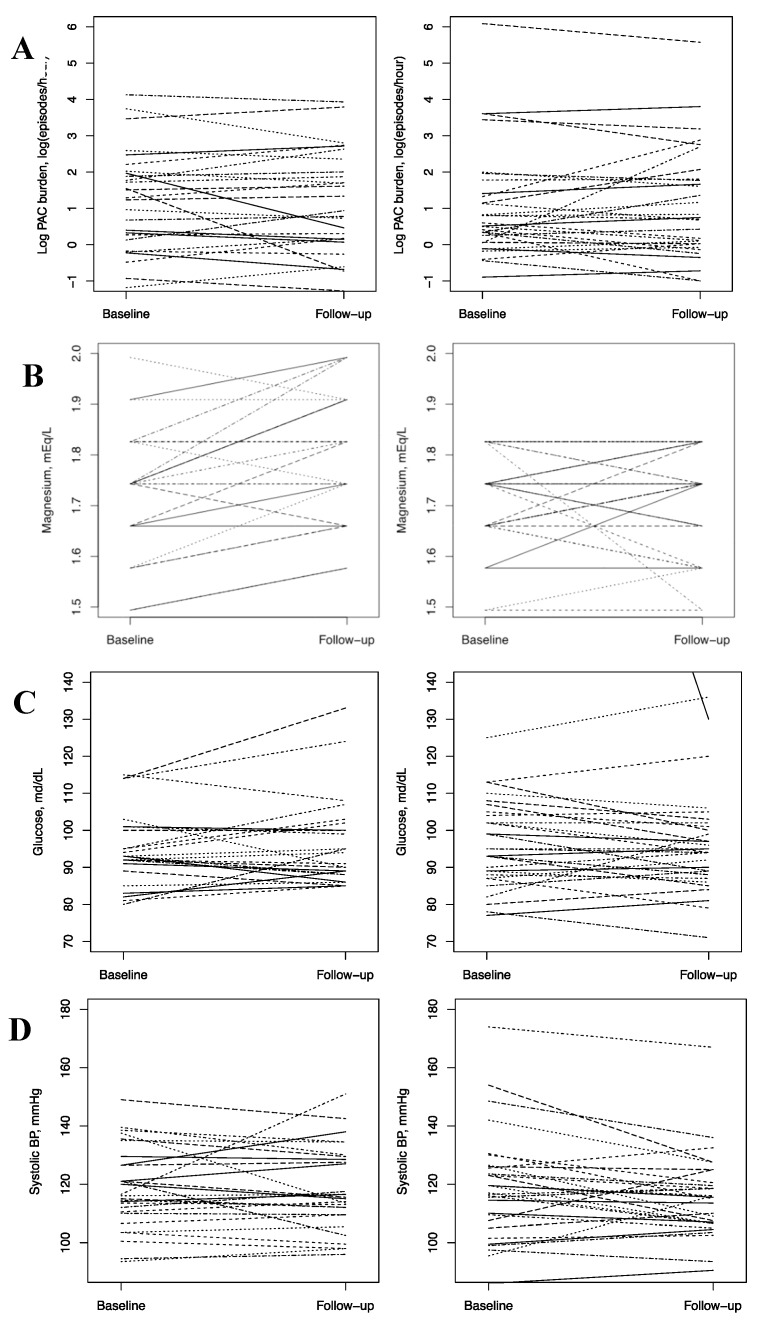
Spaghetti plots for change in (**A**) log PAC burden, (**B**) change in serum magnesium, (**C**) serum glucose *, and (**D**) SBP (systolic blood pressure). * One participant had a baseline glucose concentration of 307 mg/dL. The baseline value for this participant is outside the frame. PAC, premature atrial contractions; and BP, blood pressure.

Table 3 presents the study outcome values at baseline and follow-up, as well as age- and baseline value-adjusted differences in change according to intervention assignment. Spaghetti plots depicting individual change over the intervention period are provided in Figure 2. At follow-up, the ZioPatch average wear times were similar to the baseline, with 13.0 ± 1.8 days for the intervention group, 12.7 ± 2.3 days for the placebo group, and 92.6% assigned to magnesium and 73.3% assigned to placebo wearing ≥12 days. For the primary outcome, log PAC burden (episodes per hour), change over the intervention period was −0.06 (95% confidence interval (CI): −0.33, 0.20) for those randomized to the magnesium supplement and 0.05 (95% CI: −0.23, 0.33) for those randomized to the placebo. In the multivariable-adjusted models, there was no evidence of an intervention effect; the ratio of geometric means was 0.94 (0.69, 1.3), *p*-value = 0.73. Similarly, in subgroup analyses, the effect did not differ according to baseline magnesium concentration above versus below the median (Table 4; *p*-interaction = 0.88).

Magnesium supplementation was not significantly associated with change in serum glucose (2.4 (95% CI: −3.0, 7.7) mg/dL; *p* = 0.39). The lack of association remained in sensitivity analyses, excluding one participant with extremely high baseline glucose (2.8 (95% CI: −0.9, 6.4) mg/dL) and one participant who reported changing his/her diabetes medication status during the follow-up (2.0 (95%: −3.6, 7.5) mg/dL). The intervention was also not significantly associated with change in systolic or diastolic blood pressure overall (2.9 (95%: −1.4, 7.2) mmHg and −0.5 (95%: −3.5, 2.5) mmHg, respectively) or after excluding two participants who changed their blood pressure medication status between the baseline and follow-up visits.

In post-hoc analyses, where we additionally adjusted for sex, the results were similar. Also, no meaningful patterns emerged in additional subgroup analyses by age category and sex.

### 3.5. Safety and Tolerability of the Intervention

When asked an open-ended question about adverse events, the most commonly reported responses were related to gastrointestinal (GI) symptoms. Of the intervention group, 32% commented on GI changes at intervention day 21, 30% at day 42, and 33% at day 80. In the placebo group 7% commented on GI changes at intervention day 21, 4% at day 42, and 0% at day 80. When considering unique individuals, 50% assigned to magnesium and 7% assigned to placebo commented on GI changes at any point in the study. Specific GI comments, by intervention day, are provided in Table 5.

One person in the intervention group experienced side effects, which led the participant to discontinue blinded study treatment.

At the end of the study, when asked to which group they were assigned, among those assigned to the active treatment, 15% guessed magnesium supplements, 14.3% guessed placebo, and 35.7% reported not knowing (15 participants did not respond). Of those assigned to the placebo, 4.3% guessed magnesium supplements, 26.1% guessed placebo, and 69.6% reported not knowing (7 participants did not respond).

## 4. Discussion

In this pilot trial of 59 relatively healthy adults aged 55 and older, supplementation with 400 mg of magnesium daily over 12 weeks was safe and well tolerated and led to a change of 0.07 mEq/L in serum magnesium, which is substantial enough in magnitude that in a larger sample size it may translate to health outcomes. The intervention was not associated with change in PACs, but estimates of association had wide confidence intervals, and the study was not powered to identify important differences. Likewise, there was no association between supplemental magnesium and changes in glucose, systolic blood pressure, or diastolic blood pressure.

The mechanisms through which magnesium supplementation could reduce the risk of supraventricular arrhythmias and AF are not fully understood. However, magnesium is known to play a direct role in cardiac contractility [21]. Small studies in healthy individuals and in patients with cardiac disease have found that intravenous magnesium administration prolongs sinoatrial, intra-atrial, and atrioventricular node conduction and the atrial refractory period, which in turn may contribute to prevent the onset of AF [22,23,24]. Also, randomization to 148 mg of oral magnesium (and 296 mg of potassium) intake (vs. the placebo) had antiarrhythmic effects among 232 patients with frequent ventricular arrhythmias [25].

Blood pressure and diabetes are also established risk factors for AF [26,27], through which magnesium may lower AF risk. In the present pilot trial, changes in blood pressure and serum glucose did not differ significantly for those given the magnesium supplementation and those given the placebo. This is in contrast with the existing literature; however, our study was small, and confidence intervals around the treatment differences were wide. Meta-analyses of randomized controlled trials have consistently demonstrated that magnesium supplementation lowers blood pressure in a dose-dependent manner [28,29,30]. In the most recent meta-analysis, a median dose of 368 mg/d for a median duration of 3 months significantly reduced systolic blood pressure (BP) by 2.0 mm Hg (95% CI: 0.4, 3.6) and diastolic BP by 1.8 mm Hg (95% CI: 0.7, 2.8) [30]. Based partly on this evidence, in November 2016, a petition was filed with the Food and Drug Administration (FDA) for a qualified health claim for magnesium and reduced risk of high blood pressure (FDA-2016-Q-3770). A comparable meta-analysis of RCTs, including a total of 370 patients with type 2 diabetes, found that magnesium supplementation (median dosage 360 mg/day) reduced concentrations of fasting blood glucose (−10.1 mg/dL, 95% CI −19.8, −0.2) over a median intervention duration of 13 weeks [31]. These meta-analyses suggest that magnesium is causally related to hypertension and abnormal glucose homeostasis. However, their interpretation is complicated by the fact that the individual studies included in the meta-analyses were highly heterogeneous in terms of magnesium formulation and dosage and participant characteristics.

In terms of serum magnesium, the intervention of 400 mg of magnesium oxide daily was associated with a serum increase of 0.07 mEq/L. This finding is concordant with results from a meta-analysis of the effect of magnesium supplementation dosage on serum magnesium response. In the meta-analysis the median dose was 360 mg of magnesium/day, the intervention length was 12 weeks, and the response was 0.08 mEq/L [32]. In the meta-analysis there was evidence of an inverse relationship between the baseline magnesium concentration and responsiveness to the supplementation. A similar phenomenon was not observed in the present trial; however, in our sample, the baseline magnesium concentrations were quite high, and power was exceedingly low for subgroup comparisons.

The results from this study provide additional evidence about compliance with magnesium supplementation at the dosage of 400 mg of magnesium daily, as well as safety and tolerability. Among the participants randomized to magnesium, only 1 out of 29 participants (3.5%) ceased the intervention due to side effects. The compliance in this study was good, at 75% in the intervention group and 83% in the placebo group, according to pill counts. The low drop-out rate and high compliance provides support for the tolerability of this dosage. However, the fact that 50% in the intervention group commented on GI changes at some point in the follow-up should not be dismissed. Unfortunately, given the way side effects were assessed, it is not possible to quantify the severity of the GI complaints. Notably, several individuals only commented about GI changes in the first few days after taking the study treatment.

The primary limitation of this study is the small size, which led to an imbalance of some key potential confounding factors, such as sex. Among those randomized to magnesium, 86.2% were female, whereas among those randomized to the placebo, 60.0% were female. This is important, because AF risk is known to be greater among men [3]. However, the findings were similar in post-hoc sensitivity analyses where we adjusted for sex. An additional consideration is that the baseline serum concentrations of the trial participants were quite high; it is unclear how serum magnesium would have changed in a context of low baseline magnesium concentrations or how that may translate to change in other physiologic outcomes. Lastly, we assessed tolerability with a simple open-ended question, not a checklist of specific signs and symptoms graded for severity according to a standard toxicity table.

## 5. Conclusions

In sum, this small pilot double-blinded randomized controlled trial of supplementation with 400 mg of magnesium daily provides evidence to support the safety and tolerability of this intervention and for adherence to the ZioPatch heart rhythm monitoring device. Despite our study population being largely magnesium replete, a change in serum magnesium was observed. Magnesium supplementation was not associated with change in PACs, glucose, or blood pressure; however, this small pilot study was short-term and not powered to identify small-to-moderate clinically relevant differences. The results of this pilot study will guide the design of a larger trial to evaluate the effect of supplemental magnesium on arrhythmias.

## Figures and Tables

**Table 1:** Baseline participant characteristics * overall and stratified by intervention status.

	Overall	Magnesium (400 mg Daily)	Placebo	*p*-Value
*N*	59	29	30	
Demographics				
Age, years	61.5 ± 5.2	61.3 ± 5.3	61.6 ± 5.2	0.814
Age category				0.761
≥65 years	14 (23.7)	6 (20.7)	8 (26.7)	
<65 years	45 (76.3)	23 (79.3)	22 (73.3)	
Sex				0.039
Female	43 (72.9)	25 (86.2)	18 (60.0)	
Male	16 (27.1)	4 (13.8)	12 (40)	
Race				0.612
White	56 (94.9)	27 (93.1)	29 (96.7)	
Nonwhite	3 (5.1)	2 (6.9)	1 (3.3)	
Educational attainment				0.279
High school graduate or GED	1 (1.7)	0 (0)	1 (3.3)	
Some college	10 (16.9)	6 (20.7)	4 (13.3)	
College graduate	26 (44.1)	10 (34.5)	16 (53.3)	
Graduate school or professional school	22 (37.3)	13 (44.8)	9 (30)	
Physiologic characteristics				
Height, cm	167.9 (9.2)	167.1 (8.1)	168.7 (10.3)	0.491
Weight, kg	79.2 (18.2)	78.0 (18.0)	80.5 (18.7)	0.603
BMI, kg/m^2^	27.9 ± 4.6	27.7 ± 4.9	28.0 ± 4.5	0.804
Serum magnesium, mEq/L	1.72 ± 0.11	1.74 ± 0.12	1.71 ± 0.10	0.308
Systolic blood pressure, mmHg	119 ± 16	119 ± 14	119 ± 18	0.933
Diastolic blood pressure, mmHg	71 ± 9	72 ± 9	71 ± 10	0.627
Antihypertensive medication	14 (24)	9 (31)	5 (17)	0.233
Serum glucose, mg/dL	98.9 ± 29.9	94.2 ± 10.6	103.2 ± 40.2	0.242
Sensitivity analysis **	95.2 ± 11.1	94.2 ± 10.6	96.2 ± 11.6	0.494
Glucose lowering medication	2 (3.4)	0 (0)	2 (6.7)	0.492
PAC burden, episodes/h	14.5 ± 58	8.5 ± 14	20.2 ± 80	0.437
Median (25th, 75th percentiles)	2.28 (1.22, 6.86)	3.64 (1.31, 7.57)	1.75 (1.12, 4.01)	
Log PAC burden, log(episodes/h)	1.15 ± 1.42	1.26 ± 1.35	1.04 ± 1.49	0.566
Median (25th, 75th percentiles)	0.82 (0.20, 1.92)	1.29 (0.27, 2.02)	0.55 (0.11, 1.39)	

GED, general education diploma; BMI, body mass index; PAC, premature atrial contractions; and SD, standard deviation. * mean ± SD or *n* (%). ** Omission of one participant with a baseline glucose value of 307 mg/dL.

**Table 2:** Self-reported compliance.

**Compliance**	**% Reporting Missing Pills**			**Reason for Missing Pills, *N***			
				**Forgot**	**Too Busy**	**Makes Me Sick**	**Other**
Ever reported *							
Magnesium		60%		7	0	5	2
Placebo		52%		11	2	0	3
	**% Reporting Missing Pills in:**			**Reason for Missing Pills, *N***			
Reported at Specific Follow-Up Visits **	**Last 3 Days**	**Last 1 Week**	**Last 2 Weeks**				
Intervention Day 21							
Magnesium	8%	12%	20%	2	0	2	1
Placebo	0%	7%	24%	7	1	0	0
Intervention Day 42							
Magnesium	22%	33%	39%	5	0	5	1
Placebo	15%	22%	31%	6	2	0	2
Intervention Day 80							
Magnesium	14%	27%	40%	5	0	1	0
Placebo	12%	23%	28%	6	1	0	1

* Over any time frame (i.e., last 3 days, last 1 week, last 2 weeks). ** Responses not mutually exclusive (e.g., the same individual could have reported forgetting to take pills at intervention days 21, 42, 80).

**Table 3:** Change in PACs and secondary endpoints (i.e., systolic blood pressure (SBP), diastolic blood pressure (DBP), serum glucose, serum magnesium) according to treatment group.

	Magnesium (400 mg Daily) Mean (SD)	Placebo Mean (SD)	Intervention Effect Coefficient * (95% CI)	*p*-Value
**Primary outcome (episodes/h)**				
Log PAC burden			0.94 (0.69, 1.3) **	0.73
Baseline	1.26 (1.4)	1.04 (1.49)		
Follow-up ^†^	1.16 (1.41)	1.09 (1.53)		
Change	−0.06 (0.68)	0.05 (0.75)		
PAC burden			0.44 (−2.58, 3.46)	
Baseline	8.5 ± 14	20.2 ± 80		
Follow-up ^†^	8.1 ± 12	14.6 ± 48		
Change	−0.6 ± 7	−5.6 ± 33		
**Secondary outcomes**				
Serum magnesium, mEq/L			0.07 (0.03, 0.12)	0.002
Baseline	1.74 (0.12)	1.71 (0.1)		
Follow-up ^‡^	1.8 (0.13)	1.71 (0.11)		
Change	0.07 (0.09)	0 (0.1)		
Serum glucose, mg/dL				
Baseline	94.2 (10.6)	103.2 (40.2)	2.4 (−3.0, 7.7)	0.39
Follow-up ^‡^	96.3 (12.2)	96.2 (13.7)		
Change	1.8 (7.5)	−7.1 (32.8)		
Serum glucose ^¥^, mg/dL				
Baseline	94.2 (10.6)	96.2 (11.6)	2.8 (−0.9, 6.4)	0.14
Follow-up ^‡^	96.3 (12.2)	95 (12.4)		
Change	1.75 (7.5)	−1.21 (6.7)		
Systolic blood pressure, mmHg			2.9 (−1.8, 7.2)	0.18
Baseline	119 (14)	119 (18)		
Follow-up ^‡^	118 (14)	115 (14)		
Change	−1 (10)	−4 (10)		
Diastolic blood pressure, mmHg			−0.5 (−3.5, 2.5)	0.74
Baseline	71.8 (8. 7)	70.6 (10.3)		
Follow-up ^‡^	71.0 (8.8)	70.8 (8.7)		
Change	−0.5 (7.1)	0.2 (6.1)		

CI, confidence Interval; DBP, diastolic blood pressure; PAC, premature atrial contractions; SD, standard deviation; and SBP, systolic blood pressure. * Adjusted for age (≥65 or <65), and baseline concentration (e.g., when change in glucose is the outcome, models were adjusted for baseline glucose). The numbers of observations included in linear models are 57, 57, 54, 54, 53, 58, and 58 for the outcomes log PAC burden, PAC burden, serum magnesium, serum glucose, serum glucose excluding outlier, systolic blood pressure and diastolic blood pressure, respectively. ** Presented as a ratio of geometric means (i.e., exp(coefficient)). ^†^ ZioPatch was worn for a 2-week period, from the follow-up clinic visit (intervention week 10) through the end of the study (intervention week 12). ^‡^ Follow-up information obtained at clinic visit (intervention week 10). ^¥^ Outlier removed.

**Table 4:** Change in PACs and secondary endpoints (i.e., SBP, DBP, serum glucose, serum magnesium) according to treatment group, stratified by baseline serum magnesium concentration.

Primary Outcome	Baseline Serum Magnesium Concentration				*p*-Interaction
	<Median		≥Median		
	Intervention Effect Coefficient * (95% CI)	*p*-Value	Intervention Effect Coefficient * (95% CI)	*p*-Value	
Log PAC burden	0.89 (0.51, 1.54) **	0.67	0.91 (0.61, 1.35) **	0.64	0.88
Serum magnesium, mEq/L	0.05 (0, 0.10)	0.04	0.12 (0.04,0.20)	0.004	0.24
Serum glucose, magnesium/dL	−4.7 (−13.3, 4.0)	0.29	6.0 (2.0, 10.0)	0.03	0.06
Serum glucose, magnesium/dL ^¥^	−3.2 (−9.0, 2.6)	0.28	6.0 (2.0, 10.0)	0.03	0.01
Systolic blood pressure, mmHg	4.8 (1.0, 8.5)	0.01	3.8 (−2.5, 10.2)	0.24	0.96
Diastolic blood pressure, mmHg	5.5 (0.6, 10.4)	0.03	2.4 (−5.6, 0.8)	0.14	0.009

CI, confidence Interval; DBP, diastolic blood pressure; PAC, premature atrial contractions; and SBP, systolic blood pressure. * Adjusted for age (≥65 or <65) and baseline concentration (e.g., when change in glucose is the outcome, models were adjusted for baseline glucose). ^¥^ Outlier removed. ** Ratio of geometric means.

**Table 5:** Gastrointestinal (GI)-related responses to the open-ended question, “Since starting the study, have you experienced anything out of the ordinary?” *.

Intervention Day #	Intervention	Comment
**Day #21**	Magnesium	Less solid stools
	Magnesium	Initially, I took the pill before bed with calcium and fish oil and a blood pressure med. It did not really make me sick, but I felt some bloating and cramping. I switched to taking it in the am, and that works better. That was the reason for missing.
	Magnesium	Diarrhea
	Magnesium	My stools have changed in consistency and color.
	Magnesium	I have had some diarrhea but that could be due to my innards. They have been unpredictable since my abdominal/colorectal surgeries.
	Magnesium	After 4 pills, I quit taking them due to intestinal issues. I was in the bathroom the third and fourth day and very crampy all day. I emailed and was told I could quit taking them.
	Magnesium	The first two days, I experienced brief bouts of diarrhea about 90 min after taking the pills. No problems since.
	Magnesium	Some diarrhea and gas
	Placebo	Sudden onset of nausea lasting about 30 s about an hour after taking the pill.
	Placebo	Increase in diarrhea but could be from the increase in nuts in my diet.
**Day #42**	Magnesium	Slightly often stools
	Magnesium	Diarrhea
	Magnesium	Slight nausea, slight pain in stomach, increased flatulence
	Magnesium	Upset Stomach
	Magnesium	The initial 4 pills made me sick. Also, I am currently stressed as my (spouse) is scheduled for (major) surgery next week.
	Magnesium	A little diarrhea an hour or so after taking the pill, but this only happened on the first two days.
	Magnesium	Had gastrointestional issues when taking the pill.
	Magnesium	Loose stools, some diarrhea, and cramps after taking pill in the morning.
	Placebo	Diarrhea, but could be due to increased nut intake,
**Day #80**	Magnesium	Some difficulty with digestion
	Magnesium	My fingernails have gotten must stronger, and my bowels were loose and somewhat sluggish.
	Magnesium	I have been a lot ‘looser’ since taking the pills.
	Magnesium	Upset stomach
	Magnesium	Small bouts of diarrhea the first two days of taking the pills; nothing since.

#, number .* Some details were modified slightly to reduce the likelihood of identifying a participant. Minor spelling and punctuation changes were made to improve clarity.
